# Development and validation of Adaptability to Return-to-Work Scale (ARTWS) for cancer patients

**DOI:** 10.3389/fpsyg.2023.1275331

**Published:** 2024-04-29

**Authors:** Yu-Jie Guo, Ping Xue, Wen-wen Gu, Xiao-qin Su, Jia-mei Li, Ben-xin Kuai, Jia-shuo Xu, Hui-wen Xie, Ping-ping Han

**Affiliations:** ^1^School of Nursing and Rehabilitation, Nantong university, Nantong, Jiangsu, China; ^2^Office of Joint Medicine, Taizhou Second People’s Hospital, Jiangyan District, Taizhou, Jiangsu, China; ^3^Department of Neurosurgery, Rudong People’s Hospital, Nantong, Jiangsu, China

**Keywords:** cancer patient, adaptability of return-to-work, validation, development, scale

## Abstract

**Introduction:**

The research on cancer patients returning to work in China is still in its infancy, and there is no research and discussion on the adaptability to return-to-work for cancer patients. It is critical to develop the Adaptability to Return-to-Work Scale (ARTWS) for cancer patients and evaluate its psychometric properties.

**Methods:**

The items of the initial scale were compiled based on the theoretical model and literature review results. Through two rounds of Delphi expert consultation (*N* = 15) and a pilot survey (*N* = 40), the initial scale was further checked and revised. Conduct a large sample survey (*N* = 376) and the construct validity and reliability of the ARTWS were assessed by confirmatory factor analysis (CFA) and exploratory factor analysis (EFA).

**Results:**

The final ARTWS consisted of 24 items. “Focusing on rehabilitation,” “Rebuilding Self-efficiency,” and “Adjusting plans” as common factors in determining adaptability to return to work for cancer patients, and the cumulative variance contribution rate for these three factors was 66.6%. The S-CVI of the total scale was 0.979. The Cronbach’s α coefficient was 0.937 and the 2-week test–retest reliability was 0.814.

**Discussion:**

ARTWS has good correlation validity and can be used as a tool to measure the adaptability of cancer patients’ return to work. The presentation of the manuscript in Research Square (https://doi.org/10.21203/rs.3.rs-2323264/v1).

## 1 Introduction

Given the developments in cancer control and treatment regimes, the age-standardized 5-year relative survival increased for most cancer types, and the number of long-term survivors has also steadily increased (Zeng et al., 2018). It is noteworthy that approximately 40–50% of cancer patients worldwide are of working age at the time of diagnosis (Klaver et al., 2020). Studies have shown that cancer often leads to employment-related problems such as absenteeism, unemployment, reduced income, and early retirement (Mehnert et al., 2013; Osowiecka et al., 2020). The loss of employment has a significant effect on the affected individual and may lead to financial problems, decreased quality of life, and low self-esteem (Seifart and Schmielau, 2017). Studies have shown that return to work plays an important role in a cancer patient’s life by structuring everyday life and strengthening the identity (Isaksson et al., 2016). Additionally, return to work improves the quality of life and provides satisfaction related to work (Rasmussen and Elverdam, 2008; Knott et al., 2014; Stone et al., 2017). Therefore, it is important to study various aspects of cancer patients’ return to work.

Cancer patients often face physical, psychological, and social maladjustments and a lack of coping resources when they return to work (Knott et al., 2014). Mehnert (2011) proposed that cancer survivors’ lack of physical energy, psychological ineptness, anxiety, and depression are related to the reduction of working time after returning to work. In addition, cancer survivors may experience discrimination at work, such as being forced to quit or denied promotion, or the inability to obtain health insurance. Adaptation to cancer is a continuing process where a patient attempts to manage emotional suffering, solve specific cancer-related problems, and gain command or control over life events related to the disease (Barroilhet Diez et al., 2005). Good coping skills and adaptability of cancer patients are positive personal factors for returning to work (Stergiou-Kita et al., 2014). Thus, it is important to help cancer patients improve their adaptability to return to work.

Through a systematic review of job-related assessment tools in the field of cancer, six tools were found: Return-To-Work Self-Efficacy Questionnaire (Lagerveld et al., 2010), 19-item return-to-work self-efficacy (Shaw et al., 2011), Readiness for Return-To-Work Scale (Franche et al., 2007), Lam Assessment of Stages of Employment Readiness (Franche and Krause, 2002), World Health Organization (1993), Successful Return-To-Work Questionnaire for Cancer Survivors (Greidanus et al., 2020; Appendix A). However, the main purpose of these six assessment tools is to assess the patient’s return to work belief, status, or ability to work. So far, there are no assessment tools for cancer patients’ return to work adjustment. Therefore, to measure the adaptability of cancer patients to return to work and explore their sustainability, it is necessary to develop a special scale to evaluate the adaptability of cancer patients to return to work.

So, our study aimed to construct the self-reported Adaptability to Return-to-Work Scale (ARTWS) for cancer patients with good reliability and validity.

## 2 Materials and methods

### 2.1 Theoretical framework

A theoretical model of “Cancer patients’ return-to-work adaptation experience and coping resources” was constructed by our team (Xu et al., 2023) based on the grounded theory, which defines the adaptability to return-to-work for cancer patients as the ability of cancer patients to mobilize internal and external resources to rebuild themselves and cope positively in the face of physiological, psychological, and social complexities during the process of returning to work and the ability to respond positively. In the model, the adaptability to return to work for cancer patients is divided into three stages: focusing on recovery, rebuilding efficiency, and adjusting planning. And two categories of resources: personal resources and external resources.

### 2.2 Procedure

The ARTWS was developed in two stages. The first stage was the development of tools. The items of the initial scale were compiled according to the theoretical model based on interviews and analysis of 30 cancer patients who returned to work in the previous stage and a literature review constructed by the research group. Then the initial scale was further evaluated and revised through two rounds of Delphi surveys and a pilot survey. Stage 2 has conducted a cross-sectional survey of cancer patients, which was conducted to evaluate the psychometric properties of the ARTWS.

#### 2.2.1 Stage 1: developing the initial scale for ARTWS

##### 2.2.1.1 Delphi method

A two-round Delphi survey among a panel of experts was used to generate consensus on the content of the preliminary scale. The scale items were revised based on the experts’ scores on the assessment of the importance of the scale items (using the scores on the Likert scale: 1 = not important, 5 = very important) and the feedback they provided in the open-ended “Revision Comments” column, as well as the results of the group discussion. Through the coefficient of expert (Cr) to the expert’s evaluation of the research content of the degree of reliability, calculated as Cr = (Cs + Ca)/2, where Cs represents the expert’s familiarity with the content of this study, Ca represents the expert to give the evaluation of the basis of judgment. It is generally believed that Cr ≥ 0.7 means that the expert has a high authority in the field of this study, and the results obtained by consulting the expert are more reliable. The degree of coordination of experts’ opinions was assessed by calculating Kendall’s W. The larger the value of Kendall’s W, the better the degree of coordination of experts’ opinions and the higher the consistency (Henney et al., 1982).

##### 2.2.1.2 Pilot study

Forty cancer patients followed in the oncology chemotherapy-radiotherapy departments of two hospitals in Nantong were selected for the pre-survey. After the patients completed the first version of the scale, we assessed whether they were able to understand each item correctly and listened to their comments on the content and expression of the scale.

#### 2.2.2 Stage 2: psychometric evaluation of ARTWs

##### 2.2.2.1 Participants

A cross-sectional study was conducted in four hospitals located in Jiangsu Province, China. The Affiliated Hospital of Nantong University, the First People’s Hospital of Nantong, the Third People’s Hospital of Nantong, and the Third People’s Hospital of Rugao participated in the study. Subjects were recruited between September 2020 and October 2021. Four trained research assistants contacted the staff to identify the potentially eligible patients. The inclusion criteria were: (1) diagnosis of cancer by pathological examination; (2) ≥18–60 years old; (3) being aware of the diagnosis; (4) working at the time of diagnosis; (5) completion of treatment and in the follow-up period in stable condition with complete or partial remission, as evaluated a specialist; (6) could read and write in Chinese; and (7) volunteered to participate in this study. Patients with mental disorders or cognitive handicaps and patients with stage 4 tumors were excluded.

##### 2.2.2.2 Questionnaires and sample size

The questionnaire included participants’ demographic data (age, sex), medical data (pathology, stage), and self-reported ARTWS. Medical data were obtained from the patient’s case information record system to ensure accuracy.

The recommended sample size is 10 respondents per survey item (Boateng et al., 2018), hence this study predicted the need for 200–300 study participants; 400 questionnaires were eventually distributed in this study.

##### 2.2.2.3 Correspondence of experts

The criteria for the selection of experts were: ① engaged in oncology clinical care, psychology, and other fields of work or scientific research; ② familiar with the test methods of scale psychometrics; ③ more than 10 years of research work or scientific research in the field related to the topic; ④ intermediate and above titles; ⑤ bachelor’s degree and above; and ⑥ voluntary participation in the study, and a high degree of motivation for the research in the field of psychosocial oncology.

##### 2.2.2.4 Ethical considerations

This study was approved by the Human Research Ethics Committee of the Affiliated Hospital of Nantong University, Jiangsu, China (Project No.202065). All procedures performed in this study were by the ethical standards of the institutional and/or national research committees and with the 1964 Helsinki Declaration. All participants signed an informed consent form.

### 2.3 Statistical analysis

#### 2.3.1 Item analysis

Standard deviation and coefficient of variation were used to measure the differentiation of items. Standard deviation ≥0.75 and coefficient of variation ≥0.15 were used as the standard. Large standard deviation and coefficient of variation indicated good discriminability of items. The change in Cronbach’s α coefficient of the scale was evaluated after deleting items one by one. If Cronbach’s α coefficient increased after deleting an item, it indicated that the behavior or psychological trait measured by the item was different from that measured in other items. A value less than 0.4 indicates that this item is not homogenous with other items. Critical ratio (CR) was adopted to test the discrimination and differentiation of the items. Items with a CR value lower than 3 or with no statistically significant difference (*p* > 0.05) were deleted (Ye et al., 2018).

#### 2.3.2 Construct validity

Exploratory factor analysis (EFA), the correlation coefficient between each dimension, and the correlation coefficient between a dimension and the total scale were used for construct validity. The Kaiser–Meyer–Olkin (KMO) measure and Bartlett’s test of sphericity were used to ensure that the data had sufficient inherent correlations to perform EFA. Bartlett’s test of sphericity was considered significant at *p* < 0.05 and KMO value >0.8, which then justified the use of EFA (Yang et al., 2019). Confirmatory factor analysis (CFA) was used to assess the factorial structure extracted from EFA. Measurement models were tested using IBM Amos software version 24.0 with maximum likelihood estimation. Common goodness-of-fit indices were calculated to assess CFA and incremental fit indices such as the CFI and IFI (values >0.90 indicated a good fit). The root mean square error of approximation (RMSEA) needed to be <0.8, and the chi-square divided by the df value was considered good at <3 (Shahsavari et al., 2020; Yi et al., 2020).

#### 2.3.3 Content validity

The content validity index of the total scale (S-CVI) and the content validity index of each item (I-CVI) were calculated according to the expert evaluation opinions. The content validity index at the scale level was expressed as the content validity index of the average scale level (S-CVI/AVE). It is generally believed that the content validity is good when I-CVI is above 0.78, and S-CVI/AVE is greater than 0.9 (Liu et al., 2020).

#### 2.3.4 Reliability

Cronbach’s α coefficient was used to reflect the internal consistency of the scale and each facet. An alpha value >0.70 was considered acceptable (Xu et al., 2021). Thirty patients were retested at an interval of 2 weeks. The test–retest reliability was assessed by calculating the Pearson correlation coefficient (or Spearman rank correlation coefficient for non-normal distribution) between the two measurements to determine the stability of the scale. If the correlation coefficient of two-week test–retest reliability was greater than 0.7, good reliability was indicated (Muramatsu et al., 2019). Interclass Correlation Coefficient (ICC) is a metric that reflects both the extent of correlation and the consistency between measurements. The value of ICC ranges from 0 to 1, with a better consistency being indicated if the score is ultimately greater than 0.75.

## 3 Results

### 3.1 Analysis of the Delphi survey results

An expert team of 15 specialists was invited from Zhejiang and Jiangsu provinces, including 10 oncology nurses, 2 oncologists, 1 psychologist, and 2 oncology rehabilitation physicians, to screen and assess the scale items. The average work experience of the experts varied from 6 to 36 (20.73 ± 8.61) years. In the Delphi study, the expert authority coefficient of the two rounds of expert consultation was 0.85, and the questionnaire recovery rate was 100%. In the first round of consultation Kendall’s *W* = 0.297, *p* < 0.05 of the chi-square test; in the second round of consultation Kendall’s W = 0.514, *p* < 0.05, which indicates that the experts’ opinions converge and the degree of harmonization is high (see Table 1). Ultimately, 10 items were deleted, 1 item was added, and 2 items were merged into 1 to derive a second version of the scale containing 25 items.

**Table 1:** The basic information of specialists and Cr.

Code	Gender	Age	Education attainment	Specialist field	Length of employment	*Cs*	*Ca*	*Cr*
1	Woman	56	Bachelor’s degree	Oncology clinical nursing management	36 years	0.80	0.88	0.84
2	Woman	40	PhD degree	Oncology clinical nursing management	12 years	0.80	0.90	0.85
3	Woman	43	PhD degree	Research in oncology nursing	10 years	0.80	1.00	0.90
4	Woman	43	Master’s degree	Oncology clinical nursing management	25 years	0.80	0.90	0.85
5	Woman	38	Master’s degree	Oncology clinical nursing	17 years	0.60	0.90	0.75
6	Woman	39	PhD degree	Research in oncology nursing	17 years	0.80	0.90	0.85
7	Woman	51	Master’s degree	Oncology clinical nursing management	30 years	1.00	0.90	0.95
8	Woman	49	Bachelor’s degree	Oncology clinical nursing	24 years	0.80	0.80	0.80
9	Man	50	Master’s degree	Oncology clinical care	25 years	1.00	1.00	1.00
10	Woman	37	Bachelor’s degree	Oncology clinical nursing	15 years	0.60	0.80	0.70
11	Woman	47	PhD degree	Oncology clinical nursing management	23 years	1.00	0.90	0.95
12	Man	55	Master’s degree	Psychological research	30 years	0.80	0.88	0.84
13	Woman	37	Bachelor’s degree	Oncology clinical nursing	13 years	0.60	0.80	0.70
14	Woman	38	Bachelor’s degree	Oncology clinical nursing	6 years	0.60	1.00	0.80
15	Man	47	Bachelor’s degree	Oncology clinical care	28 years	1.00	0.80	0.90
Mean value						0.80	0.89	0.85

### 3.2 Patient characteristics

In this study, a total of 400 questionnaires were sent out and 376 completed questionnaires were received with a recovery rate of 94.0%. There were no missing values in the returned questionnaire. This investigation covered the common cancer types in China, including lung cancer, breast cancer, gynecological cancer, digestive system cancer, head and neck cancer, and prostate cancer, with the majority of the cases being breast cancer. EFA was performed on the first 176 cases. The patients ranged in age from 25 to 60 (47.20 ± 8.87) years. CFA was performed on the remaining 200 cases. The patients ranged in age from 20 to 60 (47.91 ± 9.42) years old (see Table 2) for other details.

**Table 2:** The basic information of patients for EFA and CFA.

Characteristics	Classification	EFA frequency (percentage)	CFA frequency (percentage)
Gender	Male	38 (21.60%)	55 (27.50%)
	Female	138 (78.40%)	145 (72.50%)
Marital status	Married	164 (93.18%)	183 (91.50%)
	Unmarried/Divorced/Death of spouse	12 (6.82%)	17 (8.50%)
Education	Primary school education or below	6 (3.41%)	25 (12.50%)
	Junior high school	45 (25.57%)	75 (37.50%)
	Senior high school	43 (24.43%)	53 (26.50%)
	Bachelor’s degree	80 (45.45%)	42 (21.00%)
	Master degree or above	2 (1.14%)	5 (2.50%)
Medical insurance	Without health insurance	1 (0.57%)	6 (3.00%)
	Urban medical insurance	163 (92.61%)	167 (83.5%)
	Rural insurance	12 (6.82%)	27 (13.5%)
Cancer type	Breast cancer	115 (65.34%)	94 (47.00%)
	Cancer of digestive system	30 (17.05%)	58 (29.00%)
	Respiratory cancer	10 (5.68%)	20 (10.00%)
	Cancer of reproductive system	10 (5.68%)	18 (9.00%)
	Others	11 (6.25%)	10 (5.00%)
Cancer staging	I	39 (22.16%)	72 (22.16%)
	II	82 (46.59%)	80 (46.59%)
	III	55 (31.25%)	48 (31.25%)

### 3.3 Item analysis

There were no items in the second version of ARTWS that showed the floor or ceiling effects. However, the standard deviation of item A1 “I monitor my health status as instructed” was less than 0.75, and the “corrected total item correlation” of the same was less than 0.4, indicating that its dispersion degree and homogeneity with other items were not ideal. The item A1 in the deletion scale cannot enlarge the Cronbach’s α coefficient. Hence, this item was excluded after the panel discussion. After a comprehensive analysis, 24 items were retained.

### 3.4 Content validity

Six nursing experts working in the field of cancer for more than 10 years were selected to evaluate the scale items using a 5-point scale (see Table 3). We revised the questionnaire items according to the experts’ advice. Some items were also revised based on the participating patients’ comments. The I-CVI scores ranged from 0.833 to 1.00 for all items, all above 0.78 (Cui et al., 2017). The S-CVI/AVE was computed as 0.979, which is within the acceptable range (see Table 4).

**Table 3:** Basic information of 6 experts.

Code	Gender	Age	Education attainment	Specialist field	Length of employment
1	Woman	40	PhD degree	Oncology clinical nursing management	12 years
2	Woman	43	PhD degree	Research in oncology nursing	10 years
3	Woman	43	Master’s degree	Oncology clinical nursing management	25 years
4	Woman	38	Master’s degree	Oncology clinical care	17 years
5	Woman	39	PhD degree	Research in oncology nursing	17 years
6	Man	55	Master’s degree	Psychological research	30 years

**Table 4:** Content validity of the scale.

Item	Expert number						I-CVI	S-CVI
	1	2	3	4	5	6		
B9	5	5	4	5	5	5	1.000	0.979
B8	5	5	5	5	5	5	1.000	
B7	5	5	5	5	5	5	1.000	
B13	5	5	5	5	5	5	1.000	
B6	5	5	5	5	5	5	1.000	
B10	5	5	5	5	5	5	1.000	
C1	5	5	5	5	5	5	1.000	
B14	5	5	4	4	4	4	1.000	
C7	5	5	4	4	3	4	0.833	
C5	5	5	4	4	4	4	1.000	
C2	5	5	5	5	5	4	1.000	
C8	5	5	4	4	3	5	0.833	
B12	5	5	4	4	3	5	0.833	
C4	5	5	4	4	4	4	1.000	
C9	5	5	4	4	4	4	1.000	
A9	5	5	4	4	4	5	1.000	
B18	5	5	4	4	4	4	1.000	
B11	5	5	5	5	5	5	1.000	
A3	5	5	5	5	5	5	1.000	
A7	5	5	5	5	5	5	1.000	
A6	5	5	5	5	5	5	1.000	
A4	5	5	5	5	5	5	1.000	
A5	4	5	4	4	5	4	1.000	
A2	4	5	4	4	5	4	1.000	

A, Focus on rehabilitation; B, Rebuilding self-efficacy; C, Adjusting plans.

Numbers after the ABC letters represent the order of entries under each dimension in the initial version of the scale.

### 3.5 Construct validity

The Bartlett’s sphericity test result of the scale was <0.001, and KMO was 0.882, which was suitable for EFA. The factors with an eigenvalue greater than 1 were extracted by principal component analysis. The maximum coefficient of variation method was used for orthogonal rotation (Varimax) to obtain the results of the factor load matrix after rotation. Three common factors were generated, and the cumulative variance contribution rate was found to be 66.6%. The factor loading of 24 items ranged from 0.476 to 0.910, all of which were greater than 0.4, without multiple loading. A total of 24 items were finally retained in the formal scale, including 9 items of Adjusting plans (factor 1), 9 items of Rebuilding self-efficacy (factor 2), and 6 items of Focusing on rehabilitation (factor 3) (see Tables 5, 6). In addition, the correlation coefficient between each factor ranged from 0.349 to 0.520, and between each factor and the total scale from 0.671 to 0.854 (see Table 7). The results of the ICC revealed that the mean of single rater/measurement was 0.812 (*p* < 0.05), which indicates this scale has better consistency.

**Table 5:** Factor load matrix based on exploratory factor analysis of ARTWS.

Items	Factor 1	Factor 2	Factor 3
B9 I can adapt changes in the work after return-to-work	**0.859**	0.217	0.132
B8 I will arrange my workload after return-to-work according to my health condition	**0.855**	0.207	0.140
B7 I will adjust my work goals after return-to-work according to my health condition	**0.829**	0.138	0.246
B13 I will take the initiative to negotiate with my employer on return-to-work issues	**0.781**	0.320	0.174
B6 I can anticipate the stress of return-to-work	**0.773**	0.081	0.103
B10 I will maintain a balance between work and health	**0.761**	0.226	0.260
C1 I can maintain the peace of mind, when faced with difficulties encountered in return-to-work	**0.691**	0.248	0.166
B14 I will take the initiative to seek help, when encounter with difficulties in return-to-work	**0.685**	0.308	0.167
C7 I can find comfort and motivation in faith	**0.476**	0.172	0.343
C5 I can get my family’s encouragement and support for return-to-work	0.143	**0.845**	0.066
C2 I can get support and care from my supervisor for return-to-work	0.231	**0.836**	0.094
C8 I can get advice and help from my peers for return-to-work	0.165	**0.825**	0.099
B12 I can derive confidence to return-to-work from my past experience in overcoming difficulties	0.218	**0.823**	0.110
C4 I can get the role model support from fellow patients who have returned to work successfully	0.152	**0.751**	0.013
C9 I can get return-to-work guidance from medical staff	0.072	**0.733**	0.255
A9 I’m eager to return-to-work	0.259	**0.703**	0.131
B18 I believe I can return to a normal family and social life by return-to-work	0.350	**0.669**	0.180
B11 I think I have a responsibility to return-to-work	0.185	**0.537**	0.149
A3 I will avoid the factors that are detrimental to my rehabilitation	0.173	0.104	**0.910**
A7 I can dissolve kinds of bad emotions in time	0.134	0.123	**0.896**
A6 I can effectively deal with kinds of physical discomfort	0.109	0.143	**0.887**
A4 I can keep a healthy lifestyle	0.291	0.119	**0.766**
A5 I can do rehabilitation exercises actively	0.268	0.109	**0.762**
A2 I reflect on the cause of my illness seriously	0.177	0.177	**0.648**

Factor 1, Adjusting plans; Factor 2, Rebuilding self-efficacy; Factor 3, Focusing on rehabilitation.

A, Focus on rehabilitation; B, Rebuilding self-efficacy; C, Adjusting plans.

Numbers after the ABC letters represent the order of entries under each dimension in the initial version of the scale.

The bolded values indicate that the factor loadings are >0.400, and can be classified as one type of dimension in the rotated factor loading matrix.

**Table 6:** Total variance explained.

**Items**	**Eigen**			**% of Variance (unrotated)**			**% of variance (rotated)**		
	Eigen value	% of variance	Cumulative % of variance	Eigen value	% of variance	Cumulative % of variance	Eigen value	% of variance	Cumulative % of variance
Adjusting plans (Factor 1)	10.153	42.304	42.304	10.153	42.304	42.304	5.767	24.030	24.030
Rebuilding self-efficacy (Factor 2)	3.273	13.638	55.942	3.273	13.638	55.942	5.661	23.586	47.616
Focusing on rehabilitation (Factor 3)	2.557	10.655	66.597	2.557	10.655	66.597	4.555	18.981	66.597

Consistency. The Cronbach’s α coefficient of the total scale was 0.937 and for each factor was 0.919–0.930.

Reliability. The test–retest reliability of each factor ranged from 0.714 to 0.881 and that of the total scale 0.814, both of which were statistically significant (*p* < 0.05).

**Table 7:** The correlation between each factor and the total scale.

Items	Adjust planning (Factor 1)	Rebuilding self-efficacy (Factor 2)	Focus on rehabilitation (Factor 3)	Total scale
Adjusting plans (Factor 1)	1.000			
Rebuilding self-efficacy (Factor 2)	0.520**	1.000		
Focusing on rehabilitation (Factor 3)	0.475**	0.349**	1.000	
Total scale	0.833**	0.854**	0.671**	1.000

**p < 0.05.

**Figure 1:**
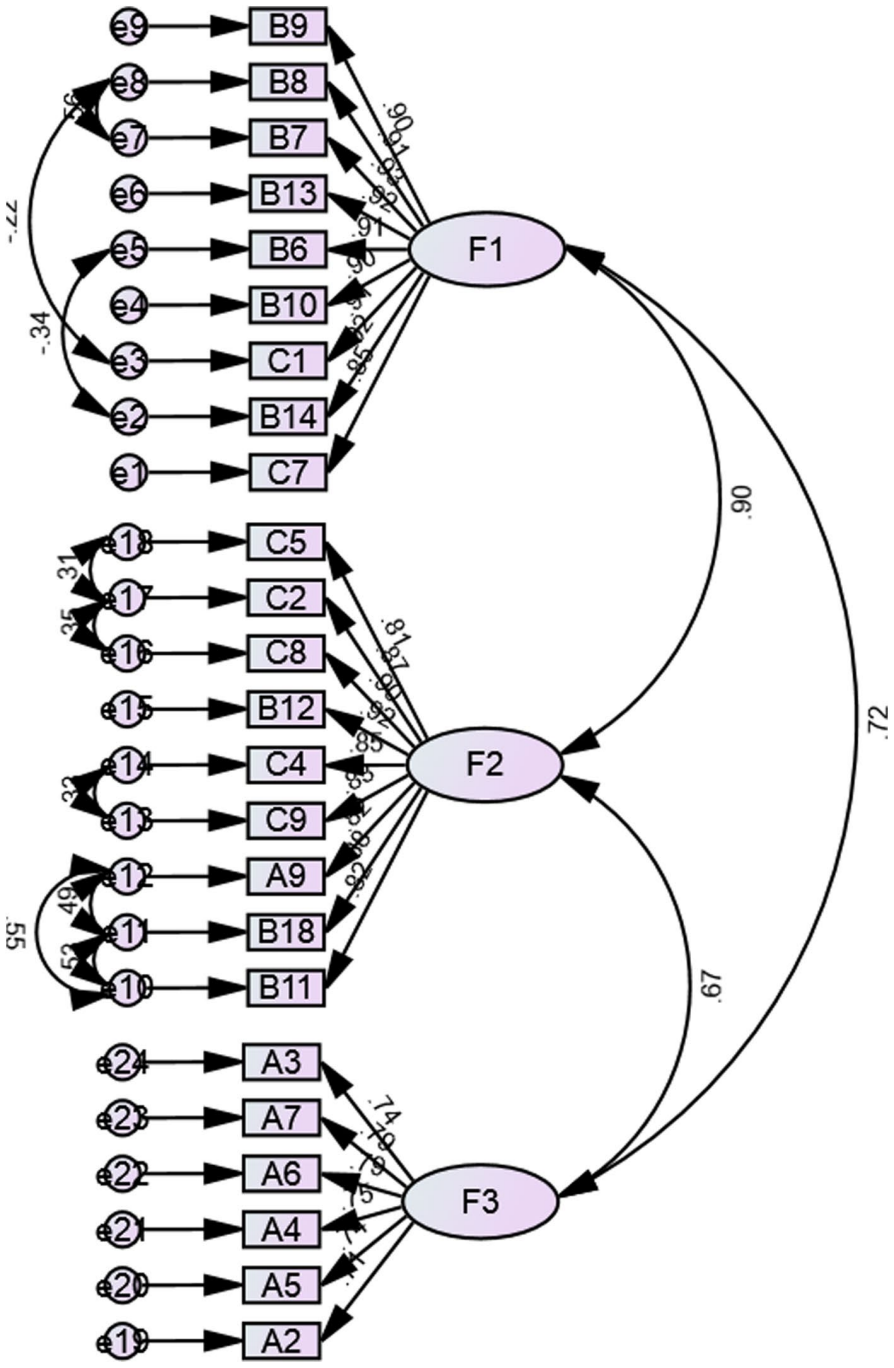
Final CFA model of the ARTWS.

We assessed the factorial structure extracted from EFA by using maximum likelihood CFA. The results of model fitting showed that CMIN/DF = 3.085, RMSEA = 0.102, RMR = 0.047, CFI = 0.910, IFI = 0.911, TLI = 0.900. CMIN/DF and RMSEA do not meet the ideal standards. Based on the modification indices, several paths of covariance between error and items were added to achieve an improved fitting model, resulting in CMIN/DF = 1.970, RMSEA (90% CI) = 0.70, CFI = 0.959, IFI = 0.959, TLI = 0.953. Figure 1 shows the final model obtained from CFA.

## 4 Discussion

Cancer patients desiring to return to work must implement effective measures to strengthen their work adaptability, thus achieving a better return to work and continuous employment. The concept of adaptability to return to work emerged from the theoretical model constructed by our group in the early stages using grounded theory (Xu et al., 2023). Based on the fact that there is no available measurement tool to assess adaptability to return to work in cancer patients, we intended to construct a scale based on the previous research. The results showed that the scale has good reliability and validity and can be used to measure the level of adaptability of cancer patients returning to work.

We found that the adaptation of cancer patients to return to work is a process of self-reconstruction using available resources and encompasses physical, psychological, and social aspects (Islam et al., 2014; Zamanzadeh et al., 2018). In addition, support from employers, colleagues, family members, and healthcare workers has an impact on cancer survivors returning to work. The theoretical model on which this scale is constructed also pays attention to the role of external support resources. At the same time, the model paid attention to the role of personal internal support resources, such as beliefs, psychological resilience, and cognition, in the process of returning to work (Xu et al., 2023). Hence, the scale that we have compiled has strong theoretical support.

In the Delphi expert consultation, we selected 15 experts from different cancer-related fields, ensuring a good representativeness. The results of two rounds of expert consultation indicated that the expert’s authority and enthusiasm were high. The results of internal consistency and retest reliability testing show that the scale has good reliability and stability. Moreover, the analysis results of the KMO index indicate that it is suitable for factor analysis. In the EFA, only three common factors were generated, although five aspects were set out in the qualitative research. It showed that the scale had good structural validity, with a focus on rehabilitation, rebuilding self-efficiency, and adjusting plans.

The results of the CFA confirmed that after model modification, the fitting of the model was within the acceptable range. The possible explanation for this result is that the internal qualities and external support resources permeate all stages and aspects of return-to-work. For example, whether during the rehabilitation of work-related physiological functions or the process of making positive adjustments to work plans, the personal resources of cancer patients provide spiritual support. Moreover, in the early stages of return to work, family support may focus on care, which can help cancer patients recover physiologically and psychologically, while in the later stages, family support is focused on the integration of resources, which helps cancer patients in finding suitable work.

### 4.1 Limitations and implications for nursing research and practice

This study has the following limitations: (1) In this study, the criterion validity test for the scale was not conducted primarily because no existing scale closely aligned with the concept of the adaptability of cancer patients to return to work. (2) Due to the pandemic, our study was confined in scope. Nonetheless, the patients demonstrated high levels of cooperation, thanks to the dedicated efforts of our team members. Future investigations can expand to include multi-center surveys with larger sample sizes to provide a more comprehensive assessment of the scale’s reliability and validity.

Implications for nursing research and practice: (1) Using the ARTWS can help Chinese cancer patients to evaluate their adaptability to return to work and provide the basis for constructing personalized return-to-work plans. (2) Moreover, both the theoretical model and scale of this study primarily focus on Chinese cancer patients. For the development of a universal scale applicable to cancer populations in various countries, future studies should assess individuals from diverse cultural backgrounds. (3) In later study, we will conduct CFA on more patients and continue to improve and verify the applicability of the scale.

## 5 Conclusion

The ARTWS for adult cancer patients has good reliability and validity and can be used as a tool to measure the adaptability of cancer patients’ return to work. The scale needs to be applied to more patients with different types of cancer in the future to verify its applicability.

## Data availability statement

The datasets generated during and/or analyzed during the current study are available from the corresponding author on reasonable request.

## Ethics statement

All procedures performed in studies involving human participants were in accordance with the ethical standards of the institutional and/or national research committee and with the 1964 Helsinki Declaration and its later amendments or comparable ethical standards. The study was approved by the Human Research Ethics Committee of the Affiliated Hospital of Nantong University (No. 202065). Informed consent was obtained from all individual participants included in the study.

## Author contributions

Y-JG: Conceptualization, Funding acquisition, Investigation, Resources, Supervision, Writing – review & editing. PX: Data curation, Methodology, Writing – review & editing. W-wG: Data curation, Methodology, Writing – review & editing. J-mL: Conceptualization, Data curation, Writing – original draft. X-qS: Formal analysis, Conceptualization, Methodology, Data curation, Writing – review & editing. B-xK: Conceptualization, Formal analysis, Writing – review & editing. J-sX: Writing – review & editing. H-wX: Writing – review & editing. P-pH: Writing – review & editing.

## Appendix A

Summary table of the return-to-work assessment tools for cancer patients.

**Table:**

Evaluation tool	Developer	Theoretical basis	Scale structure	Validated in the cancer population	Main measurement content
Return-To-Work Self-Efficacy Questionnaire (RTW-SE)	Lagerveld et al.	Based on the self-efficacy theory of the Bandura	Individual dimensions, 11 items	Yes	Measure patients’ beliefs about their ability to deal with multiple issues of physical support, cognition, emotions, relationships, and professional competence. And the scale was suitable for both before and after returning to work.
19-item return-to-work self-efficacy (RTWSE-19)	Shaw et al.	Based on the results of previous focus group interviews by Shaw and Huang	Three dimensions, Meet the job requirements (7 items), Adjust the work tasks (7 items), Request to others (5 items)	Yes	Measure patients’ concerns, expectations, and beliefs about returning to normal work through a three-dimension test.
Readiness for Return-To-Work Scale (RRTW)	Franch et al.	Based on the return-to-work readiness model	Six dimensions, First 4 dimensions (13 items): Italian forward stage, intention, action preparation-self-assessment, action preparation-action; The last 2 dimensions (9 items): Uncertain maintenance, active maintenance	Yes	This scales includes two sections that measures patients who have not returned to work and those who have returned to work respectively, with a higher score indicating a higher belief in returning to work.
Lam Assessment of Stages of Employment Readiness (LASER)	Lam et al.	Stage change model	3 dimensions (14 items): Pre-intention period; Period of intention; Action period	Yes	Evaluating the patients’ confidence in returning to work at different stages of employment preparation to observe their employment tendency.
Work Ability Index (WAI)	The Finnish Institute of Occupational Health	Pressure-strain concept and equilibrium models	Seven items (10 questions in total)	Yes	Measure the work ability.
Successful Return-To-Work Questionnaire for Cancer Survivors (I-RTW_CS)	Greidanus et al.	Based on the results of the focus group interviews of cancer survivors	Individual dimensions, Seven items	Yes	Measure Patients’ perceptions of their successful return to work by their enjoyment of their work, their health effects, and their interactions with employers.
